# Changes over Time in COVID-19 Vaccination Inequalities in Eight Large U.S. Cities

**DOI:** 10.1177/23780231231161045

**Published:** 2023-03-16

**Authors:** S. Michael Gaddis, Colleen M. Carey, Nicholas V. DiRago

**Affiliations:** 1 NWEA, Portland, OR, USA; 2 University of California, Los Angeles, Los Angeles, CA, USA; 3 Cornell University, Ithaca, NY, USA

**Keywords:** COVID-19, vaccine, community, inequality, disparities, socioeconomic status

## Abstract

The authors estimate the associations between community socioeconomic composition and changes in coronavirus disease 2019 (COVID-19) vaccination levels in eight large cities at three time points. In March, communities with high socioeconomic status (SES) had significantly higher vaccination rates than low-SES communities. Between March and April, low-SES communities had significantly lower changes in percentage vaccinated than high-SES communities. Between April and May, this difference was not significant. Thus, the large vaccination gap between communities during restricted vaccine eligibility did not narrow when eligibility opened up. The link between COVID-19 vaccination and community disadvantage may lead to a bifurcated recovery whereby advantaged communities move on from the pandemic more quickly while disadvantaged communities continue to suffer.

In early 2021, state and local authorities in the United States vaccinated millions of individuals weekly against coronavirus disease 2019 (COVID-19). By late April, more than 80 million people, about one quarter of the U.S. population, were fully vaccinated, reducing their risk for symptomatic and asymptomatic infection, transmission, hospitalization, and death. In many urban areas, vaccine doses were scarce through April. Vaccine eligibility progressed in stages, starting with health care workers and proceeding, per state and local policy, to individuals of advanced age, in certain occupations, or with particular comorbidities. By May, vaccine supply approached demand in more places, and 44 states and the District of Columbia had expanded vaccine eligibility to everyone 16 years and older (Howard 2021).

We may expect that the response and recovery period of the COVID-19 pandemic has differentially affected individuals and communities on the basis of existing socioeconomic status (SES) disadvantage. As others have noted, populations facing disadvantage prior to a major public health crisis fare worst both during and after the crisis (DeBruin, Liaschenko, and Marshall 2012). To date, evidence from the COVID-19 pandemic suggests a similar story. First, neighborhoods and communities with higher levels of socioeconomic disadvantage were hardest hit during the earliest stages of the pandemic. Incidences of infection and mortality have been higher where low-SES individuals and people of color constitute more of the population (Wrigley-Field et al. 2021). Second, researchers have documented community inequalities in COVID-19 vaccinations by neighborhood disadvantage during early restricted vaccination eligibility periods (DiRago et al. 2022).

It is unclear, however, how much the recovery of disadvantaged neighborhoods has lagged after restricted vaccination eligibility periods. In this research, we examine whether existing gaps in vaccination rates between advantaged and disadvantaged neighborhoods closed as vaccine eligibility expanded. We examine this issue using vaccination data from eight cities over three time points between March 21 and May 3, 2021, capturing the onset of widespread eligibility. Our findings contribute to a rapidly growing body of literature examining inequities both due to the pandemic and as a result of the response and recovery phases.

In Figure 1A, we present adjusted predictions for percentage vaccinated in each period. In March, low-SES communities (23.01 percent; 95 percent confidence interval [CI] = 20.25 percent to 25.76 percent) had significantly lower percentage vaccinated than high-SES communities (34.73 percent; 95 percent CI = 32.19 percent to 37.27 percent). In April, low-SES communities (35.79 percent; 95 percent CI = 31.55 percent to 40.00 percent) had significantly lower percentage vaccinated than high-SES communities (51.65 percent; 95 percent CI = 48.44 percent to 54.85 percent). In May, low-SES communities (45.65 percent; 95 percent CI = 40.49 percent to 50.81 percent) had significantly lower percentage vaccinated than high-SES communities (60.46 percent; 95 percent CI = 57.82 percent to 63.10 percent).

**Figure 1.**
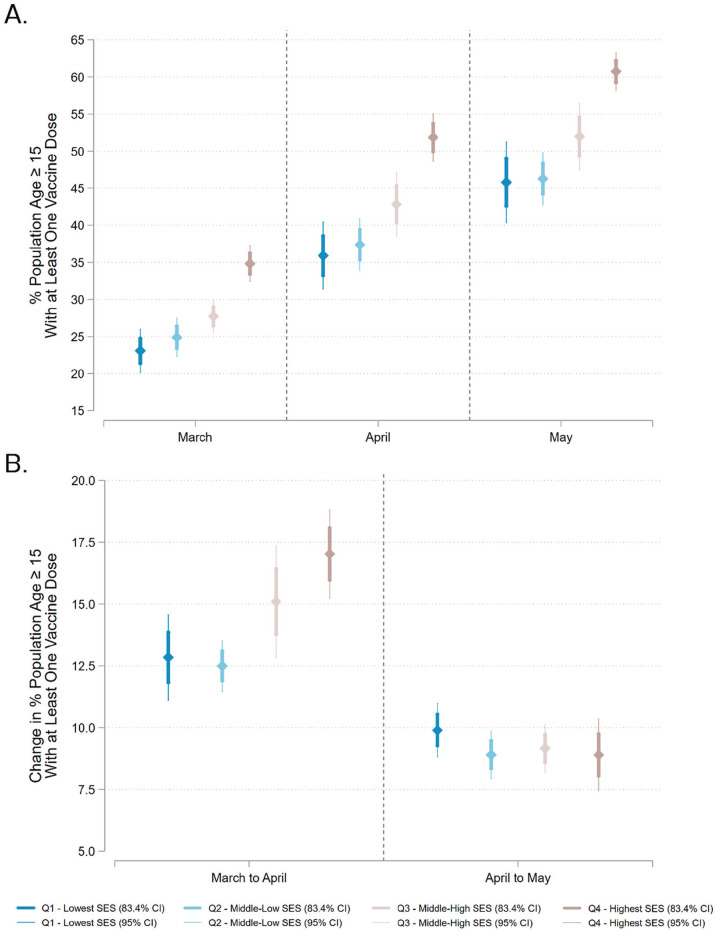
Estimated coronavirus disease 2019 vaccination and change in vaccination by ZIP code population SES composition: (A) adjusted predictions for ZIP codes with a given socioeconomic composition and (B) changes over time in adjusted predictions for ZIP codes with a given socioeconomic composition. *Note*: Color-coded points show the estimated percentage, thin lines show the 95 percent confidence interval (CI), and thick lines show the 83.4 percent confidence interval. Alongside traditional 95 percent CIs, we show 83.4 percent CIs because these visually show significant differences at the *p* < .05 level with no overlap (Cumming 2009). We estimated three population-weighted linear regressions with each time point’s vaccine rate as the dependent variable and all American Community Survey variables listed in the supplemental material as covariates. We then used the margins command in Stata/MP to estimate adjusted predictions at the means and adjusted predictions for four quartiles of socioeconomic status (SES) (Q1–Q4). We defined SES levels by setting all four SES variables to the same within-city quartiles within each scenario. We set other independent variables to within-city averages in each scenario.

In Figure 1B, we present adjusted predictions for the change in percentage vaccinated over time. Between March and April, low-SES communities (12.78 percent; 95 percent CI = 11.19 percent to 14.38 percent) had significantly lower change in percentage vaccinated than high-SES communities (16.92 percent; 95 percent CI = 15.38 percent to 18.45 percent). Between April and May, the difference between change in percentage vaccinated in low-SES communities (9.86 percent; 95 percent CI = 8.77 percent to 10.96 percent) and high-SES communities (8.82 percent; 95 percent CI = 7.39 percent to 10.25 percent) was not significant.

The percentage vaccinated in low-SES communities lagged that in high-SES communities in March, April, and May. Additionally, the large gap in percentage vaccinated between communities during the restricted vaccine eligibility period did not narrow when eligibility opened up in late April and early May. During the six weeks captured in our data, 64.5 million people received their first doses of vaccine, equal to 31.2 percent of all vaccinated individuals as of September 1, 2021. Thus, despite the rapid and widespread reach of vaccinations during this period, large inequalities persisted.

Our work suggests that a process of cumulative disadvantage at the community, and likely individual, level is unfolding because of the COVID-19 pandemic. The same communities that suffered the highest burdens of infection and mortality from COVID-19 before vaccines were available had lower levels of community vaccination during restricted vaccine eligibility and did not immediately close those gaps as eligibility opened up (Clouston, Natale, and Link 2021; Ransome et al. 2021). The link between COVID-19 vaccination and community disadvantage is concerning. Importantly, this continuing inequality may lead to a bifurcated recovery whereby advantaged communities move on from the pandemic more quickly while disadvantaged communities continue to suffer.

## Supplemental Material

sj-docx-1-srd-10.1177_23780231231161045 – Supplemental material for Changes over Time in COVID-19 Vaccination Inequalities in Eight Large U.S. Cities Click here for additional data file. Supplemental material, sj-docx-1-srd-10.1177_23780231231161045 for Changes over Time in COVID-19 Vaccination Inequalities in Eight Large U.S. Cities by S. Michael Gaddis, Colleen M. Carey and Nicholas V. DiRago in Socius
