# Supplementary material for: Changes over Time in COVID-19 Vaccination Inequalities in Eight Large U.S. Cities
Source: Socius. 2023 Mar 16;9:23780231231161045. doi: 10.1177/23780231231161045 (PMC10020851; doi:10.1177/23780231231161045)
Supplement: sj-docx-1-srd-10.1177_23780231231161045 – Supplemental material for Changes over Time in COVID-19 Vaccination Inequalities in Eight Large U.S. Cities [file sj-docx-1-srd-10.1177_23780231231161045.docx]

**DATA AND METHODS SUPPLEMENTARY INFORMATION**

We gathered official counts of the percentage of individuals with at least one dose of a COVID-19 vaccine by ZIP Codes (hereafter: communities) in eight of the ten most populous U.S. cities: New York, Chicago, Houston, Phoenix, Philadelphia, San Antonio, San Diego, and Dallas. We collected these data at three time points: March 21, April 12, and May 3, 2021. The denominator we use to calculate vaccination rates in each community is the American Community Survey (ACS) value for population age 15 and older. To account for community composition, we used ACS data on percent employed in “health care and social assistance” and age 65 and older (to control for early eligibility); percent enrolled in Medicaid or other means-tested public health insurance, without health insurance coverage, under the federal poverty line, and without internet access (to measure socioeconomic status [SES]); percent Black, Hispanic, Asian, and White (to control for race/ethnicity). We converted each SES variable into quartiles standardized by city.

We estimated three population-weighted linear regressions with each time point’s vaccine rate as the dependent variable and all ACS variables listed above as covariates. We then used the margins command in Stata/MP to estimate adjusted predictions at the means and adjusted predictions for four quartiles of SES communities. We defined SES levels by setting all four SES variables to the same within-city quartiles within each scenario. We set other independent variables to within-city averages in each scenario Our analyses provide the adjusted regression estimates for percent of vaccinations overall, in lowest SES, middle-low SES, middle-high SES, and highest SES communities in March, April, and May, and changes in vaccinations over time.

We use publicly available de-identified data, so our study was exempt from institutional review board approval. We followed the Strengthening the Reporting of Observational Studies in Epidemiology (STROBE) reporting guidelines for cohort studies.
